# X-linked Christianson syndrome: heterozygous female *Slc9a6* knockout mice develop mosaic neuropathological changes and related behavioral abnormalities

**DOI:** 10.1242/dmm.022780

**Published:** 2016-01-01

**Authors:** Jakub Sikora, Jennifer Leddy, Maria Gulinello, Steven U. Walkley

**Affiliations:** 1 Dominick P. Purpura Department of Neuroscience, Rose F. Kennedy Intellectual and Developmental Disabilities Research Center, Albert Einstein College of Medicine, Bronx, NY 10461, USA; 2 Institute of Inherited Metabolic Disorders, Charles University in Prague –1st Faculty of Medicine, Ke Karlovu 2, Praha 2 160 00, Czech Republic

**Keywords:** Christianson syndrome, *Slc9a6*, NHE6 protein, Female heterozygotes, X-chromosome inactivation, Mosaicism

## Abstract

Christianson syndrome (CS) is an X-linked neurodevelopmental and neurological disorder characterized in males by core symptoms that include non-verbal status, intellectual disability, epilepsy, truncal ataxia, postnatal microcephaly and hyperkinesis. CS is caused by mutations in the *SLC9A6* gene, which encodes a multipass transmembrane sodium (potassium)-hydrogen exchanger 6 (NHE6) protein, functional in early recycling endosomes. The extent and variability of the CS phenotype in female heterozygotes, who presumably express the wild-type and mutant *SLC9A6* alleles mosaically as a result of X-chromosome inactivation (XCI), have not yet been systematically characterized. *Slc9a6* knockout mice (*Slc9a6* KO) were generated by insertion of the bacterial *lacZ*/β-galactosidase (β-Gal) reporter into exon 6 of the X-linked gene. Mutant *Slc9a6* KO male mice have been shown to develop late endosomal/lysosomal dysfunction associated with glycolipid accumulation in selected neuronal populations and patterned degeneration of Purkinje cells (PCs). In heterozygous female *Slc9a6* KO mice, β-Gal serves as a transcriptional/XCI reporter and thus facilitates testing of effects of mosaic expression of the mutant allele on penetrance of the abnormal phenotype. Using β-Gal, we demonstrated mosaic expression of the mutant *Slc9a6* allele and mosaically distributed lysosomal glycolipid accumulation and PC pathology in the brains of heterozygous *Slc9a6* KO female mice. At the behavioral level, we showed that heterozygous female mice suffer from visuospatial memory and motor coordination deficits similar to but less severe than those observed in X-chromosome hemizygous mutant males. Our studies in heterozygous *Slc9a6* KO female mice provide important clues for understanding the likely phenotypic range of Christianson syndrome among females heterozygous for *SLC9A6* mutations and might improve diagnostic practice and genetic counseling by helping to characterize this presumably underappreciated patient/carrier group.

## Abstract (precis)

**Editors' choice:** *Slc9a6* knockout mice are a model of the X-linked neurodevelopmental and neurological disorder, Christianson syndrome. We show that heterozygous female mice express mosaic changes comparable to neuropathological abnormalities identified in mutant males.

## INTRODUCTION

Christianson syndrome (CS, OMIM 300243) is a rare, X-chromosome-linked neurodevelopmental and neurological disorder characterized in males by non-verbal status, intellectual disability, epilepsy, craniofacial dysmorphology with microcephaly, truncal ataxia and hyperkinesis. These core phenotypic features (present in >85% of patients) can be accompanied by secondary symptoms, such as signs of autism and behavioral abnormalities mimicking Angelman syndrome, eye movement problems, hypotonia or gastroesophageal reflux disease (for further information see Pescosolido et al., 2014). Magnetic resonance imaging studies have suggested hippocampal and/or progressive cerebellar atrophy in male CS patients (Gilfillan et al., 2008; Schroer et al., 2010; Mignot et al., 2013). Furthermore, neuropathological reports on male CS brains have demonstrated widespread neurodegeneration, including loss of Purkinje cells (PCs), dystrophic neuritic changes, gliosis and tau deposition (Christianson et al., 1999; Garbern et al., 2010).

CS develops as a result of mutations in the solute-carrier 9A6 gene (*SLC9A6*, Xq26.3; Gilfillan et al., 2008). *SLC9A6* codes a multipass transmembrane protein (NHE6) that is believed to co-regulate the luminal pH of early/recycling endosomes by its sodium (potassium)-hydrogen antiporter activity (Ohgaki et al., 2011; Kondapalli et al., 2014). A significant fraction of the reported *SLC9A6* mutations are nonsense or shift the open reading frame of *SLC9A6* and result in introduction of premature stop codons (reviewed by Pescosolido et al., 2014).

As a result of X-chromosome inactivation (XCI), female heterozygotes presumably express *SLC9A6* mutations in their cells and tissues mosaically. Although several female heterozygotes have presented with clinical symptoms reminiscent of those identified in their CS-affected male relatives (Christianson et al., 1999; Gilfillan et al., 2008; Schroer et al., 2010; Pescosolido et al., 2014), conclusive information about the range of the probably mitigated and/or variable clinical phenotype in this particular group still remains to be considered systematically.

Previous studies by us and others have demonstrated the relevance of the knockout of the murine S*lc9a6* gene (*Slc9a6* KO) for studies exploring the human CS phenotype. Our analyses of mutant *Slc9a6* KO males (*Slc9a6^−/Y^* ‘mutant’ males) and homozygous mutant *Slc9a6* KO females (*Slc9a6^−/−^* ‘mutant’ females), both of which serve as models with uniform tissue distribution of the (transcriptionally) active mutant *Slc9a6* allele, indicated late endosomal/lysosomal dysfunction characterized by intraneuronal accumulation of GM2 ganglioside and unesterified cholesterol in the amygdala and the CA3/CA4 and fascia dentata regions of the hippocampus (Stromme et al., 2011). In addition, both mutant males and mutant females expressed progressive, patterned PC degeneration associated with axonal spheroid formation. Behavioral testing in mutant males revealed mild but significantly increased locomotor activity and motor coordination deficits, suggesting further overlap with the human CS clinical condition (Pescosolido et al., 2014). Importantly, a subsequent study using *in vitro* experimental approaches in neuronal cultures derived from the *Slc9a6* KO model proposed that abnormal endosomal acidification caused by the NHE6 deficit attenuates tropomyosin related kinase B (TrkB) signaling and results in underdeveloped cortical and hippocampal neuritic arborization (Ouyang et al., 2013).

Although different in specific molecular details from the human situation, the random XCI and its propagation in the tissues of the developing embryo are replicated in mice (Deng et al., 2014). In the murine female brain, XCI topography generates intra- and inter-individual diversity that ranges from individual cells to the entire organ. Crucially, however, it was shown that specific neuronal populations in female mice can tend, as a result of the complex neurodevelopment, to be inactivated non-randomly on a functionally relevant spatial scale (Wu et al., 2014). Crucial for our studies, the murine KO model carries an insertion of the *lacZ-Neo* cassette into exon 6 of the *Slc9a6* gene (X.A5; Stromme et al., 2011). *lacZ*, which codes nuclear-targeted β-galactosidase (β-Gal) from *E.coli*, thus serves both as a mutagen that obliterates the *Slc9a6* open reading frame and as a transcriptional reporter that allows effective tracing of the cellular expression of the mutant *Slc9a6* allele. Important for utility of the β-Gal reporter, its expression patterns correspond to the endogenous expression of the protein as identified by mRNA expression studies (Kondapalli et al., 2013) and/or a specific anti-NHE6 antibody (Deane et al., 2013; Ouyang et al., 2013). As a further important prerequisite, the murine *Slc9a6* gene was not previously identified, similarly to humans (Cotton et al., 2015), among X-linked genes that escape XCI (Yang et al., 2010). Therefore, crucial for the present study, β-Gal expression can also be considered to reflect the inactivation of the wild-type (WT) *Slc9a6* allele in tissues of heterozygous *Slc9a6* KO female (*Slc9a6^−/+^*) mice.

Given the relevance of the murine model for CS and with the purpose of providing insights into the potential disease phenotype in human female heterozygotes carrying *SLC9A6* mutations, we performed a study to evaluate the neuropathology and behavioral presentation in heterozygous *Slc9a6* KO female mice. Here, we focused on delineating the mosaic patterns of abnormal intraneuronal lysosomal GM2 ganglioside accumulation in amygdala and hippocampus, characterizing the extent of cerebellar PC degeneration and comparing the range of motor coordination and cognitive deficits with the abnormalities observed in mutant *Slc9a6* KO males.

## RESULTS

### Expression of the mutant *Slc9a6* allele is mosaic in the brains of heterozygous female mice

Cellular and tissue expression of the mutant *Slc9a6* allele can be tracked by the *lacZ*-encoded β-Gal reporter. Using the histochemical X-GAL-based approach, we previously documented the brain-specific expression patterns of β-Gal in mutant males and mutant females (Stromme et al., 2011). Being aware of the limitations of the histochemical technique and aiming to assess β-Gal expression qualitatively and quantitatively in the brains of heterozygous females, we tested the utility of a specific antibody for detection of the reporter protein. To allow multi-immunofluorescence (IF) tissue-labeling experiments, we selected a specific anti-*E.coli* β-Gal antibody raised in chickens.

In order to assess the progression of disease at later ages than previously reported (Stromme et al., 2011) and to fully document the reference neuropathology resulting from uniform tissue distribution of the (transcriptionally) active mutant *Slc9a6* allele, we optimized IF staining conditions in the brains of X-chromosome hemizygous mutant males (aged 4, 20-22 and 32-34 weeks). We compared the results with negative findings in age-matched WT males (shown in the cerebellar PC layer in Fig. S1C) and WT females (data not shown). The anti-β-Gal antibody generated a nuclear staining pattern in the brains of mutant males, allowing us to confirm that the expression patterns of β-Gal by IF corresponded to those previously identified by X-GAL histochemistry (Stromme et al., 2011; Ouyang et al., 2013).

**Fig. 1.**
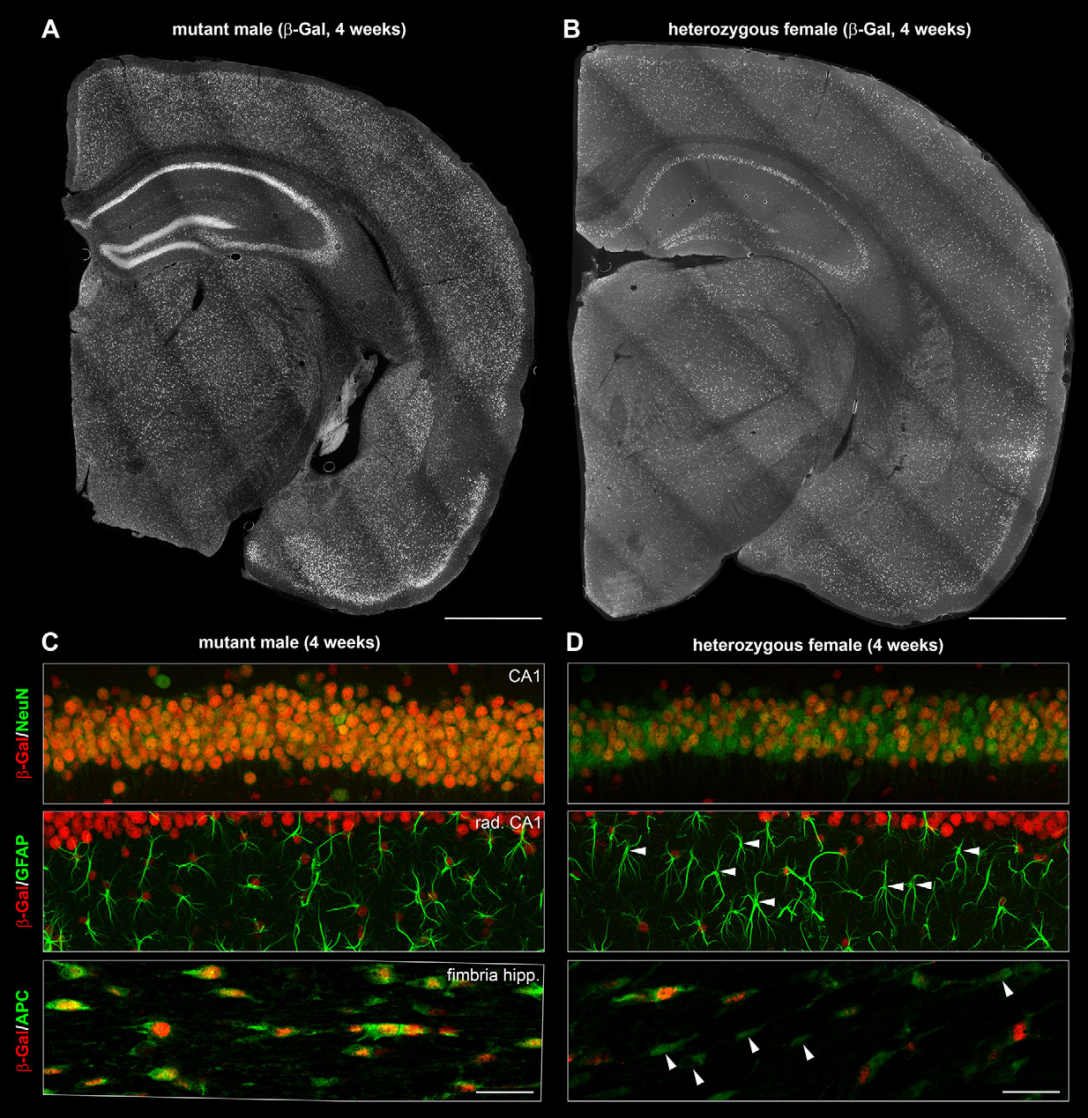
**Expression of the mutant (β-Gal^+^) *Slc9a6* allele is mosaic in heterozygous females.** Nuclear expression patterns of the β-Gal reporter in the brain of an X-chromosome hemizygous mutant male (A) are mosaically replicated in a heterozygous female (B). Unlike mutant males (C), heterozygous females express β-Gal mosaically in neurons (NeuN^+^), astrocytes (GFAP^+^) and oligondrendroglial cells (APC^+^) (D). Arrowheads depict β-Gal^−^ astrocytes and oligodendroglial cells. Cerebellar β-Gal expression in mutant males and heterozygous females is shown in Fig. 4 and Fig. S1*.* fimbria hipp., hippocampal fimbria; rad. CA1, radiatum layer of the (CA1) hippocampus. The *z*-thickness of MIP projections is 15 µm in C,D. Scale bars: 1 mm in A,B; 50 µm in C,D.

In agreement also with studies by others (Deane et al., 2013; Kondapalli et al., 2013; Ouyang et al., 2013), the versatility of the multi-IF technique allowed us to demonstrate that β-Gal expression was not limited exclusively to nuclei of neurons, but could be identified also in GFAP^+^ astrocytes and APC^+^ oligodendroglial cells (Fig. 1A,C) in the cerebra of the mutant males. As a result of the small nuclear size and relatively high levels of the endogenous cytoplasmic autofluorescence, we were not able to identify β-Gal expression unambiguously in CD68^+^ microglia/macrophages (data not shown). Crucial for progression of the neuropathological changes in mutant males, we also specifically assessed β-Gal in the cerebella and confirmed a strong expression in nuclei contained within the PC layer. Despite slight variability, β-Gal expression was uniform in PCs. Expression was also strong in the GFAP^+^ astrocytes (Bergmann glia) adjacent to PCs (Fig. S1A).

At the level of individual cells and regardless of the particular cell type, nuclear β-Gal expression was either comparable to the levels observed in mutant males or was altogether absent in *Slc9a6* heterozygous females (Fig. 1B,D; Fig. S1B). Although the overall mosaic patterning was variable between individual heterozygous female brains, it did not have a tendency to focal clustering of cells with either of the two disparate β-Gal expression profiles. Importantly, the mosaic β-Gal expression was detected in neuroanatomical locations previously identified as crucial (brain cortex, hippocampus, amygdala and cerebellar PC layer) for initiation and progression of the abnormal phenotype in mutant males.

### Intracellular accumulation of GM2 ganglioside is mosaic in heterozygous females and develops in cells expressing the mutant *Slc9a6* allele

The vacuolar intracellular GM2 ganglioside accumulation previously identified in specific neuronal populations in cerebra of mutant males and mutant females was believed to reflect late endosomal/lysosomal dysfunction. This particular subcellular pathology develops in CA3/CA4 regions of hippocampus and amygdala of these animals as early as at 3 weeks of age (Stromme et al., 2011). Utilizing the IF-based β-Gal detection, we evaluated the presence of the abnormal GM2 accumulation and correlated it with the mosaic expression of β-Gal in the brains of heterozygous females. Moreover, we assessed the timing and progression of GM2 accumulation by analyzing animals aged 4 weeks and significantly older (32-34 weeks) mutant males and heterozygous females.

**Fig. 2.**
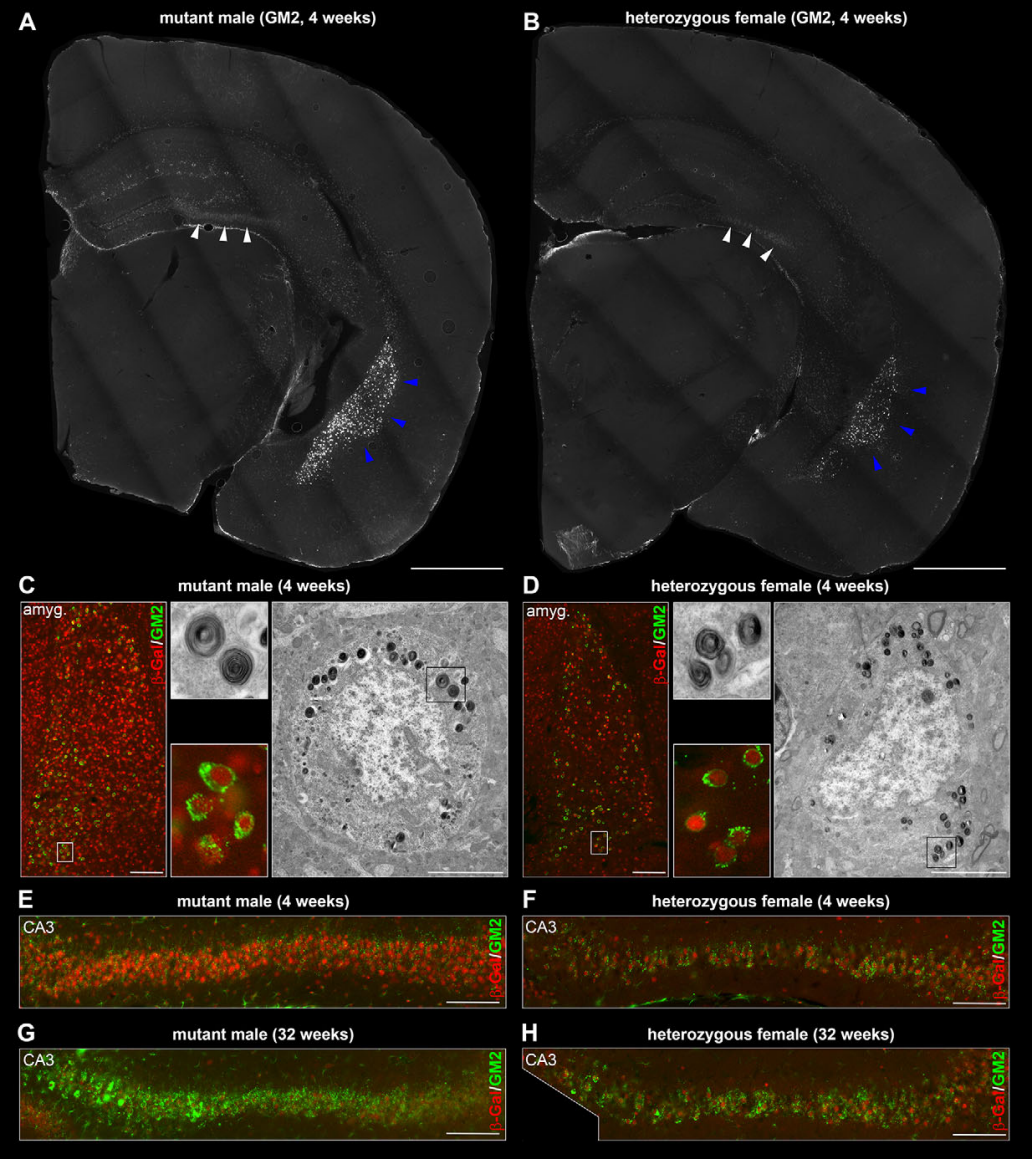
**The abnormal intraneuronal accumulation of GM2 ganglioside in amygdala and CA3 region of hippocampus is found in the neurons expressing the mutant (β-Gal^+^) *Slc9a6* allele in heterozygous females.** At 4 weeks of age, GM2 accumulation is conspicuously present in the neurons of CA3 region of hippocampus (white arrowheads) and amygdala (blue arrowheads) of mutant males (A) and in heterozygous females (B). In mutant males, only a fraction of β-Gal^+^ neurons in amygdala accumulates GM2. The intracellular glycolipid accumulation adopts the form of concentric lamellar bodies by electron microscopy (C; Fig. S2). In heterozygous females, β-Gal^+^ and β-Gal^+^/GM2^+^ neurons are less frequent than in mutant males. The lysosomal ultrastructural abnormalities found in heterozygous females (D) are equivalent to findings in mutant males. Whereas CA3 neurons in mutant males express β-Gal and accumulate GM2 uniformly (E,G), GM2 accumulation in CA3 neurons of heterozygous females is mosaic and reflects the expression of the mutant (β-Gal^+^) *Slc9a6* allele (F,H). amyg., amygdala; CA3, CA3 region of hippocampus. Scale bars: 1 mm in A,B; 200 µm (IF images) and 5 µm (electron micrographs) in C,D; 100 µm in E-H. Panels A,B showing GM2 accumulation originate from sections co-labeled for β-Gal shown in Fig. 1A,B.

Serving as a reference phenotype, mutant males showed GM2 accumulation in neurons of hippocampal CA3/CA4 regions and amygdala at 4 weeks of age (Fig. 2A). In neurons of the CA3/CA4 region of hippocampus, the nuclear β-Gal expression and GM2 (Fig. 2E) accumulation were uniform, with both signals co-occurring in individual cells. On the contrary, the number of β-Gal^+^ cells/neurons in amygdala was larger than the number of cells accumulating GM2. Nevertheless, nuclei of the GM2-accumulating neurons (Fig. 2C) consistently stained with anti-β-Gal antibody. To explore this numerical difference, we searched the amygdala of mutant males using electron microscopy to identify the abnormal glycolipid-containing concentric lamellar bodies. In agreement with the multi-IF staining, we found that a number of neurons lacked the specific lysosomal storage structures (Fig. 2C). Altogether, these observations suggest that in mutant males only a subpopulation of neurons (Fig. S2) in amygdala abnormally accumulates GM2 (and probably also other glycolipids) despite expressing the mutant *Slc9a6* allele from their single X-chromosome.

We further assessed the age-related persistence of GM2 accumulation by co-staining cerebra of significantly older mutant males (32-34 weeks). In these, we found that the patterns of β-Gal expression and the abnormal GM2 accumulation (Fig. 2G) corresponded to those identified in 4-week-old brains.

In heterozygous females (Fig. 2B,D,F,H), the neuronal GM2 pathology and its timing corresponded to the findings in mutant males. Crucially, the occurrence of GM2 accumulation was, in individual neurons, linked to mosaic nuclear expression of the β-Gal reporter. The correlation of the mosaic β-Gal expression and GM2 accumulation could easily be discerned in the hippocampal CA3/CA4 region (Fig. 2F,H). In amygdala, β-Gal expression was also mosaic (Fig. 1B; Fig. 2D). Identical to mutant males (Fig. 2C), nuclei of neurons accumulating GM2 in amygdala were also β-Gal^+^ in heterozygous females; nonetheless, some of the β-Gal^+^ cells did not accumulate GM2 (Fig. 2D).

As controls, age- and sex-matched WT littermate brains were negative for β-Gal expression and did not show intraneuronal GM2 accumulation. As an additional check for possible neurodegeneration in the regions affected by the glycolipid accumulation in mutant males and heterozygous females, we IF co-stained cerebra of animals of both these genotypes with antibodies targeting neurons (anti-NeuN) and microglia/macrophages (anti-CD68). In contrast to the cerebellum (see the section below) and when compared with WT controls, none of these stains directly or indirectly suggested widespread neurodegeneration at 4 and/or 32-34 weeks of age (data not shown).

### Purkinje cells in *Slc9a6* KO heterozygous females exhibit degeneration that is ameliorated compared with mutant males

Degeneration of PCs that is associated with spheroid formation on their axons is another feature consistently discernible in mutant males. The spatial patterning of this process follows cerebellar zonal organization with Zebrin II-positive PCs being the most resistant to decay (Stromme et al., 2011). Transformed to (para)sagittal projection (Sillitoe and Joyner, 2007), PCs in the anterior (lobules I-V) and posterior (lobule VIII) cerebellar zones tend to degenerate earlier than those localized to the central (lobule VI/VII) and nodular (lobules IX/X) zones. The molecular and developmental basis of this pattern remains to be understood fully; nonetheless, as observed in mutant males, it is replicated in other murine disease models that develop PC degeneration (e.g. Niemann–Pick disease type A/B or C; Sarna et al., 2001; Macauley et al., 2008).

**Fig. 3.**
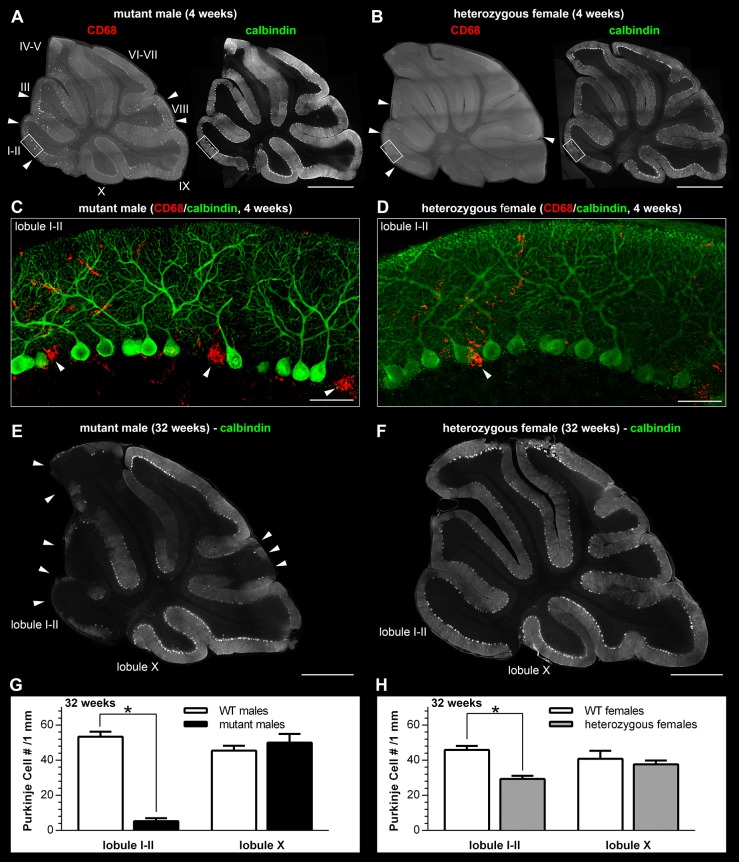
**Cerebellar pathology in heterozygous** **females.** Degeneration of Purkinje cells (visualized by calbindin IF staining) in anterior and posterior zones is initiated in mutant males (A) and heterozygous females (B) as early as at 4 weeks of age and is highlighted by the abnormal population of CD68^+^ microglia in both genotypes (arrowheads). (C,D) Neuronophagy of PC cell bodies (arrowheads) and dendrites in the molecular cerebellar layer can be readily identified at this age by confocal microscopy in both sex/genotype groups by clusters of CD68^+^ microglia/macrophages. (E) At 32 weeks of age, mutant males present with advanced and widespread loss of Purkinje cells in anterior (lobule I-V) and posterior (lobule VIII) cerebellar zones (arrowheads). (F) In heterozygous females, PC degenerative patterns are not as evident as in mutant males and vary among individual animals. (G,H) PC density quantification in lobules I-II (representative of the anterior zone) and X (representative of the nodular zone) showed significant PC loss in lobule I-II of 32-week-old mutant males and heterozygous females (*n*=4 WT and 5 mutant males and 6 WT and 17 heterozygous females) in contrast to lobule X (*n*=4 WT and 4 mutant males and 6 WT and 14 heterozygous females). *P<0.0001. The *z*-thickness of MIP projections in C,D is 15 µm, and these images correspond to areas outlined by white rectangles in A,B. Scale bars: 1 mm in A,B,E,F; 50 µm in C,D. Panel A reviews the numbering of individual cerebellar lobules.

We evaluated cerebellar pathology both in 4- and 32- to 34-week-old animals. Neurono/dendritophagic clustering of CD68^+^ macrophages/microglia in the PC and molecular layers of cerebellar lobules I-II, III and VIII (Fig. 3A,C) suggested ongoing PC loss (visualized by calbindin IF staining) in these areas already in 4-week-old mutant males. Identical but less profound changes than those identified in mutant males were also detected in 4-week-old heterozygous females (Fig. 3B,D).

At 32 weeks, cerebella of mutant males showed extensive PC loss in anterior and posterior zones (Fig. 3E). This pathology was also associated with atrophy and gliosis of the cerebellar molecular layer in these zones (shown for lobule I-II in Fig. S1A). CD68^+^ cells were frequent in these cerebellar cortical areas; nonetheless, they did not form PC neuronophagic clusters comparable to 4-week-old animals (data not shown). To document the major quantitative difference in a cohort of 32-week-old mutant males, we sampled the density of PCs in lobule I-II and lobule X selected as representative of zones affected and spared from PC degeneration, respectively. The density of PCs in lobule I-II was significantly lower in mutant compared with WT males (*F*_(1,7)_=227.4, *P*<0.0001; Fig. 3G).

Crucial for 32-week-old heterozygous females, the extent of PC degeneration was not histologically as discernible (Fig. 3F) as in mutant males. The secondary gliosis in the molecular layer of lobules affected by PC degeneration was also less profound in heterozygous females (Fig. S1B) than in mutant males. Despite these mitigated histological findings, the density of PCs in lobule I-II in heterozygous compared with WT females was significantly lower (*F*_(1,21)_=24.9, *P*<0.0001; Fig. 3H).

**Fig. 4.**
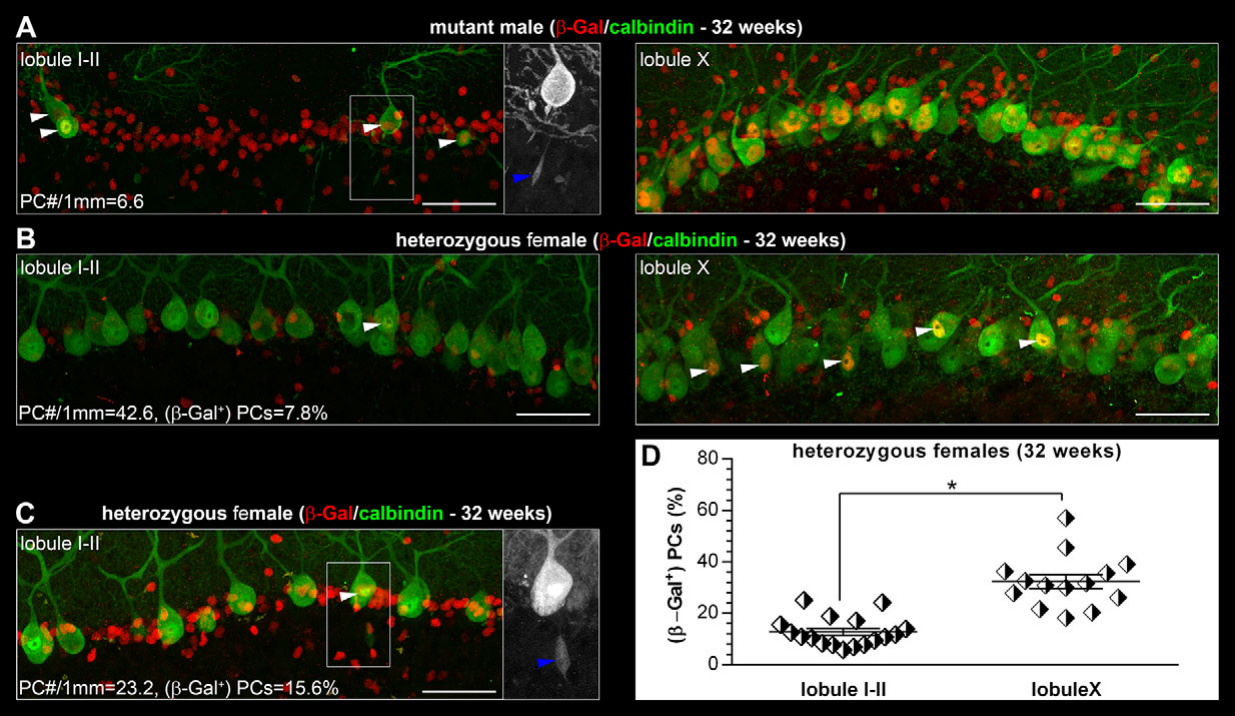
**Percentage fractions of (β-Gal^+^) PCs in 32-week-old heterozygous females differ between cerebellar lobules affected and spared from degeneration of Purkinje cells.** (A) Similar to lobule X, PCs that remain in lobule I-II uniformly express β-Gal (white arrowheads) in mutant males. Small β-Gal^+^ non-PC nuclei largely correspond to the population of (Bergmann glia) astrocytes (Fig. S1A). Axons of these remaining PCs in lobule I-II develop spheroids (blue arrowhead in the inset). (B,C) β-Gal expression in PCs is mosaic (white arrowheads) in heterozygous females and varies among individual animals. The fraction of β-Gal^+^ PCs is lower in lobule I-II compared with lobule X. β-Gal expression is also mosaic in other cell types within the PC layer (Bergmann glia astrocytes; Fig. S1B). (C) β-Gal^+^ PCs in heterozygous females also develop spheroids on their axons (blue arrowhead in the inset). (D) The percentage fraction of β-Gal^+^ PCs in lobule I-II (*n*=17) is significantly lower than in lobule X (*n*=14) of heterozygous females. *P<0.0001. Images shown for lobule I-II and X in A,B were collected from cerebella of a single mutant male and heterozygous female, respectively. Lobule I-II PC densities and percentages of β-Gal^+^ PCs in A-C represent values for the particular animals shown. The *z*-thickness of MIP projections in A-C is 18-22 µm. Scale bars: 50 µm.

Although β-Gal expression could be considered uniform in mutant males (Fig. 4A; Fig. S1A), in heterozygous females at 32 weeks of age the β-Gal expression was mosaic in the PC layer and was also confined both to PCs and to nuclei of GFAP^+^ astrocytes (Bergmann glia; Fig. S1B). Unlike mutant males, however, PC densities and the numbers of β-Gal^+^ PCs varied between individual heterozygous female cerebella (compared in two selected animals in Fig. 4B,C). To reiterate, and contrary to lobule X, the overall density of PCs was significantly lower in lobule I-II of heterozygous females when compared with their WT littermates (Fig. 3H). To quantify the likely selection against β-Gal^+^ PCs, we compared the fractional (percentage) content of β-Gal^+^ PCs in lobules I-II (mean value was 12.8±1.4%) and X (mean value was 32.4±2.8%) among heterozygous females and found that this fraction was significantly lower in lobule I-II (*F*_(1,29)_=44.6, *P*<0.0001; Fig. 4D).

As a last, but important detail, spheroid formation affected axons of (β-Gal^+^) PCs both in mutant males (Fig. 4A) and in heterozygous females (Fig. 4C).

### *Slc9a6* KO heterozygous females have cognitive deficits and sensorimotor dysfunction

**Fig. 5.**
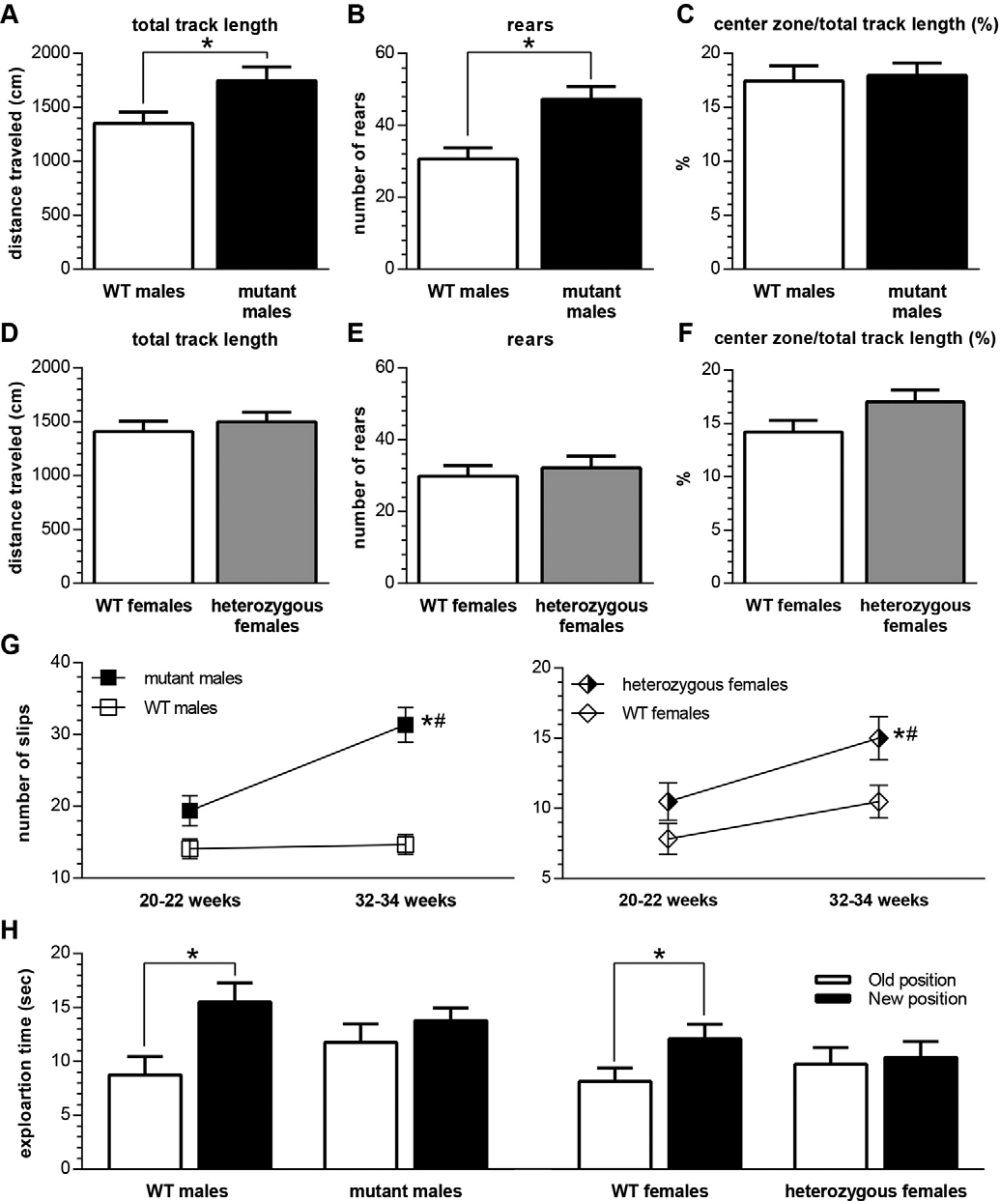
**Behavioral abnormalities in *Slc9a6* animals.** Open field (A-F) in 32- to 34-week-old animals [*n*=31 wild-type (WT) males, 30 mutant males, 22 WT females and 33 heterozygous females]. (A,B) When compared with WT littermates, mutant males demonstrated significantly higher locomotor activity (A; total track in centimeters) and exploration (B; number of rears). (C) Anxiety-like behavior was assessed as center zone exploration (center zone/total track length). (D-F) No significant differences were identified between WT and heterozygous females. *Significant differences between WT and mutant males (*P*<0.05). (G) Balance beam test in 20- to 22-week-old animals (*n*=25 WT males, 21 mutant males, 22 WT females and 34 heterozygous females) and 32- to 34-week-old animals (*n*=25 WT males, 21 mutant males, 17 WT females and 38 heterozygous females). *Significant main effect of age; ^#^significant main effect of genotype. (H) Object placement test in 20- to 22-week-old animals (*n*=15 WT males, 24 mutant males, 17 WT females and 25 heterozygous females). Both WT males and WT females had intact visuospatial memory, indicated by a clear preference (exploration in seconds) for the relocated (New position) object. On the contrary, both mutant males and heterozygous females demonstrated cognitive deficits because there was no preference for the object in the New position. *Significant difference between exploration of an object in a New and Old position (*P*<0.05). For additional details see Fig. S3.

In behavioral tests, we found that heterozygous females generally demonstrated similar motor incoordination and cognitive deficits as did male mutants, albeit some of the phenotypes were less profound in the female mice. Mutant males (32-34 weeks old) had significantly higher locomotor activity in the open field (total track length, *F*_(1,59)_=5.7, *P*<0.05; Fig. 5A) and exploration (number of rears, *F*_(1,59)_=12.5, *P*<0.05; Fig. 5B) than WT males. Similar to the previous study in younger (20- to 24-week-old) animals (Stromme et al., 2011), we identified no differences in the anxiety-like behavior (ratio of track length in center zone to total track length; Fig. 5C). Unlike the males, heterozygous and WT females at the age of 32-34 weeks did not differ in track length, rears or anxiety-like behavior (Fig. 5D-F).

Consistent with the substantial cerebellar pathology, mutant males also had significant motor coordination deficits assessed as an increased number of slips in the balance beam assay (*F*_(genotype 1,84)_=32.9, *P*<0.0001 and *F*_(age 1,86)_=11.9, *P*<0.001; Fig. 5G). Similar to the previous study (Stromme et al., 2011), gross ataxia and motor incoordination were not detectable at 20-22 weeks in mutant males but could be identified readily in animals aged 32-34 weeks. Given the mitigated cerebellar neuropathology, female heterozygotes also had significant motor coordination deficits in the balance beam compared with age-matched WT controls (*F*_(genotype 1,105)_=5.3, *P*<0.05 and *F*_(age 1,105)_=8.4, *P*<0.01; Fig. 5G). Importantly, none of the evaluated heterozygous females presented with obvious ataxia even at 32-34 weeks of age.

Given the hippocampal neuropathology of both mutant males (Stromme et al., 2011; Ouyang et al., 2013) and heterozygous females (shown in the present study), we evaluated visuospatial memory in the hippocampus-dependent object placement test (Ennaceur and Delacour, 1988; Dere et al., 2007) and found deficits in both mutant males and heterozygous females at 20-22 weeks of age (Fig. 5H). Mutant males (*T*_(d.f.=23)_=1.15, *P*<0.26) and heterozygous females (*T*_(__d.f.=24)_=0.33, *P*<0.75) failed to display a preference for a relocated object (new position not significantly greater than old position). In contrast, their age-matched WT littermates (WT males, *T*_(d.f.=14)_=5.3, *P*<0.0001; WT females, *T*_(d.f.=16)_=2.3, *P*<0.05) displayed the normal preference for a relocated object (new position significantly greater than old position). The preference for the relocated object (new position) in the testing phase was not confounded in animals of either sex by any non-specific alteration in the reactivity to novelty as assessed by the total exploration time in the training phase when no memory is involved (data provided in Fig. S3).

## DISCUSSION

Our study demonstrates that expression of the mutant *Slc9a6* allele in heterozygous female mice mosaically follows the neuroanatomical distribution and cell-type-specific patterns identified in the brains of X-chromosome hemizygous mutant males. As a consequence, heterozygous females develop behavioral and neuropathological abnormalities that are, albeit milder, comparable to defects identified in mutant males. As a follow-up to our previous studies, by analyzing animals up to 8 months old we document that specifically the sensorimotor dysfunction and the cerebellar PC degenerative pathology are progressive with age both in mutant males and in heterozygous females.

Overall, we propose that *Slc9a6* KO heterozygous female mice represent a relevant and crucial model for future studies aimed at understanding the pathogenesis of human CS. As the second pathology-expressing genotype relevant to human X-linked CS, heterozygous female mice should complement the experimental use of mutant males. As a result of the mosaic expression of the mutant *Slc9a6* allele, we expect heterozygous females to become particularly useful for testing the cell-autonomous nature of specific (neuro)pathologies and/or exploring the roles of intercellular interactions (neuronal circuitry dysfunction included) in the onset and progression of the abnormal phenotype. Similarly important will be studies aimed at exploring the embryonic and developmental impacts of XCI on overall and site-specific brain (dys)function in heterozygous female mice.

The bacterial β-galactosidase that reports the expression of the mutant *Slc9a6* allele can be identified in murine tissues by a specific antibody. With IF, we confirmed cerebral and cerebellar β-Gal expression in large populations of neurons and astrocytes (Stromme et al., 2011; Deane et al., 2013; Kondapalli et al., 2013; Ouyang et al., 2013) in mutant males and identified the mosaic presence of β-Gal in these cell types and locations in heterozygous females. Knowing the expression patterns in mutant males, presuming that the murine *Slc9a6* gene does not escape XCI (Yang et al., 2010) and comparing our results with the recently published XCI topography maps of the female murine brain (Wu et al., 2014), we explain the mosaic β-Gal expression patterns in the brains of heterozygous female mice by XCI. Previously unreported, we found β-Gal in APC^+^ oligodendrocytes of both mutant males and heterozygous females. In fact, this suggests that another glial cell type is theoretically compromised by the *Slc9a6* deficit both in the mouse model and in CS patients.

Although the neuropathological findings in human hippocampi are relatively mild (Garbern et al., 2010) and hippocampal atrophy was identified by neuroimaging studies only in some male individuals (Schroer et al., 2010), dysfunction of hippocampus and amygdala can be presumed based on the clinical CS phenotype (Stromme et al., 2011). Lysosomal accumulation of GM2 in neurons of hippocampal CA3/CA4 regions and basolateral amygdala is one of the early occurring abnormalities in *Slc9a6* mutant male mice. In the present study, we found that only a subpopulation of neurons in amygdala accumulates GM2. Molecular, structural and, most importantly, functional/connectivity characteristics (Capogna, 2014; Janak and Tye, 2015) of these neurons, the determinants of their sphingolipid pathology and its contribution to the overall phenotype remain to be established. Crucially, however, in heterozygous female mice, the timing, neuroanatomical distribution and ultrastructural appearance of GM2 pathology are replicated. As an anticipated effect, GM2 accumulation in neurons of both hippocampus and amygdala were correlated with the mosaically expressed mutant *Slc9a6* allele in heterozygous females.

The early-onset degeneration of PCs and ataxia present in older mutant male mice correspond to the clinical, neuroimaging and cerebellar pathology in male CS patients (Christianson et al., 1999; Gilfillan et al., 2008; Garbern et al., 2010; Schroer et al., 2010; Mignot et al., 2013). More specifically, PC loss and consequent atrophic changes in the cerebellar cortex of mutant males follow a previously described and conserved cerebellar developmental/gene-expression pattern (Stromme et al., 2011). In heterozygous female mice, the mosaic β-Gal expression profiles in the cerebellar cortex correspond at the cellular level to the uniform expression found in mutant males. PC degeneration in heterozygous females is initiated at the same age as in mutant males, and a selection against (β-Gal^+^) PCs is likely in heterozygous females in cerebellar cortical zones that are affected by degeneration in mutant males. Importantly, the mean values (and distribution of individual values among heterozygous females) of the percentage fraction of β-Gal^+^ PCs in the cerebellar zone(s) not affected by degeneration even in older (32- to 34-week-old) mice suggest random XCI in this particular neuronal population. This result shows that direct detection of the β-Gal reporter in the brains of heterozygous females allows *Slc9a6* allelic expression quantification even in numerically limited or anatomically restricted specific cellular types/populations. Besides neurons, we also found that GFAP^+^ cells (Bergmann glia) in the cerebellar cortex express β-Gal. Aware of the intimate developmental, structural and functional association between PCs and Bergmann glia (Bellamy, 2006; Wang et al., 2012) that encompasses regulation of synaptic transmission and/or plasticity and buffering of extracellular K^+^ and neurotrophin levels by astrocytes (Kondapalli et al., 2014), we hypothesize that the PC degeneration in mutant male or heterozygous female mice might not be an exclusively cell-autonomous event. If valid, the variable extent of XCI in PCs and cerebellar cortical astrocytes would probably, in conjunction with the microdomain and overall zonal organization of cerebellar cortex, represent a crucial determinant of the cerebellar degenerative pathology in heterozygous females.

Crucial for human CS, dominance and recessivity allelic relations valid for autosomal genes do not apply to X-chromosome-linked traits (Dobyns et al., 2004). Whereas X-linked phenotypes are penetrant in X-chromosome hemizygous male CS patients, the clinical presentation in heterozygous female patients/carriers is mitigated and often variable. One of the principal underlying mechanisms for such variability in females is XCI in their tissues (Yang et al., 2011; Deng et al., 2014). Unlike in males, data on the frequency of the mutations in X-linked genes associated with intellectual disability (ID) among females is largely unavailable as a likely result of the diversity of their clinical phenotype(s). Interestingly, it was shown that female heterozygotes in families with X-linked ID have significantly skewed XCI ratios in peripheral white blood cells (Plenge et al., 2002). However, the relation of the skewed XCI status in leukocytes to the range and severity of the neurodevelopmental, neurological and psychiatric disease(s) still remains to be understood fully (Tzschach et al., 2015). Consistent with cellular abnormalities, *Slc9a6* heterozygous female mice showed the same cognitive and sensorimotor abnormalities, albeit mitigated, that were compromised in mutant males. It remains for future studies, however, to attempt to establish neuroanatomical XCI thresholds for the behavioral pathologies and determine the sources of inter-individual variability in heterozygous female mice.

Pescosolido et al. (2014) defined the core and secondary clinical features of CS by evaluating the largest cohort of affected families and male patients. Although reported inconsistently, a variable and mitigated phenotype seems likely also in female heterozygotes for *SLC9A6* mutations. Here, some females have presented with neurodevelopmental delays, problems with speech, learning and/or behavioral difficulties, aggressiveness in childhood and adolescence, hyperkinesis or truncal ataxia (Christianson et al., 1999; Gilfillan et al., 2008; Schroer et al., 2010; Pescosolido et al., 2014). Intriguingly for interpretation of these observations, Gilfillan et al. (2008) partly excluded XCI as a contributor to the clinical CS presentation because several female heterozygotes in their pedigrees presented with normal XCI ratios in lymphocytes.

Several recent genomic projects (Tarpey et al., 2009; Schuurs-Hoeijmakers et al., 2013; Tzschach et al., 2015) demonstrated that CS is one of the most frequent X-linked neurodevelopmental ID disorders. However, the patient cohorts in these studies either consisted exclusively of male ID patients and their female relatives or included females with penetrant ID phenotypes. As a possible result of disqualifying individuals with mitigated or variable abnormalities (applicable specifically to female heterozygotes), the CS population frequency that was estimated based on some of these data sets as 1 in 16,000-100,000 (Pescosolido et al., 2014) could, in fact, be higher. Also relevant to the occurrence of CS, a substantial fraction of male CS probands develop the *SLC9A6* mutations *de novo* (Schroer et al., 2010; Pescosolido et al., 2014). This lack of maternal inheritance in CS pedigrees has not yet been specifically explored for post-zygotic mutagenic events or low-level maternal somatic/germinal mosaicism, both of which result in variable non-homogeneous tissue distribution of *SLC9A6* mutations. As these phenomena are relatively frequent in the general population (Acuna-Hidalgo et al., 2015), they could theoretically represent an additional and disparate source of phenotypic variability contributing to the likely under-diagnosis of CS.

Here, we have provided evidence for a behavioral and neuropathological phenotype in *Slc9a6* KO heterozygous female mice. Such findings, we believe, indicate a crucial need to gain detailed insight into the range of potential phenotypes in heterozygous female CS patients/carriers. Although we acknowledge that the homogenous genetic background of murine *Slc9a6* KO model might not necessarily reflect the complex and heterogeneous human genomics and X-linked epigenetics, we hypothesize that the expected XCI-driven expression (Cotton et al., 2015) of *SLC9A6* mutations in the brains of human female heterozygotes contributes to the diversity of their resultant clinical presentation. Defining the full phenotypic spectrum in this potentially underappreciated patient group is, to us, a key prerequisite to delineate the population frequency of CS, avoid diagnostic neglect and allow efficient genetic counseling and screening in affected families. Moreover, an evidence-based recognition of female CS heterozygotes as at risk or affected by a variable or mitigated disease could be an additional strong argument advocating further experimental and clinical research, including the development of therapy for this disorder.

## MATERIALS AND METHODS

### Mice and tissue collection

All procedures used in experiments involving animals were approved by the Institutional Animal Care and Use Committee of the Albert Einstein College of Medicine. The study used B6.129P2-Slc9a6^tm1Dgen/J^ (*Slc9a6* KO) mice originally acquired from the Jackson Laboratory (Bar Harbor, ME, USA). Genotyping of litters was performed as previously reported (Stromme et al., 2011). In this study, we used *Slc9a6* wild-type male (*Slc9a6^+/Y^* or ‘WT’ males) and female (*Slc9a6^+/+^* or ‘WT’ females) mice, X-chromosome hemizygous (mutant) *Slc9a6* KO male mice (*Slc9a6^−/Y^* or ‘mutant’ males) and X-chromosome heterozygous *Slc9a6* KO females (*Slc9a6^−/+^* or ‘heterozygous’ females). Homozygous (mutant) *Slc9a6* KO females (*Slc9a6^−/−^* or ‘mutant’ females) were not tested. If not stated otherwise, results were compared between genotypes and separately for sexes of the animals (mutant males×WT males and heterozygous females×WT females). For tissue collection, mice were deeply anesthetized with ketamine and xylazine and perfused transcardially with saline and subsequently with 4% paraformaldehyde (PFA). Dissected organs were further immersion fixed overnight in 4% PFA and then transferred to ice-cold phosphate buffer and stored at 4°C. This study did not use any data or material generated in our previous study (Stromme et al., 2011). Behavioral and tissue studies were performed in parallel and in identical conditions in all sex and genotype groups.

### Antibodies and immunofluorescence

For immunofluorescence (IF) labeling, brains were divided along the midline and then split into cerebral and cerebellar-brainstem parts at the mesencephalic level. The separated fragments were embedded into 8.0% sucrose and 3.5% agarose, and serial sections 35 µm thick (coronal in cerebrum and sagittal in cerebellum) were cut using a Leica VT-1000S Vibratome (Leica Microsystems, Wetzlar, Germany). Sections representing rostral-caudal bregma (−1.94 to −2.30) and lateral (0.40-0.84) ranges (Franklin and Paxinos, 2008) were selected from cerebra and cerebella, respectively. Matched sections were stained by multi-IF protocols as described before (McGlynn et al., 2004). The following primary antibodies were used for staining of specific epitopes: chicken anti-*E.coli* β-galactosidase (β-Gal) Ab (ab9361, 1:2000; Abcam, Cambridge, MA, USA), mouse anti-calbindin (Purkinje cells) mAb D-28K (C9848, 1:2000; Sigma-Aldrich, St Louis, MO, USA), rabbit anti-calbindin pAb (AB1778, 1:800; Chemicon, Temecula, CA, USA), rat anti-CD68 mAb (MCA1957, 1:1000; AbD Serotec, Kidlington, UK), mouse anti-glial fibrillary acidic protein (GFAP) mAb G-A-5 (G3893, 1:3000; Sigma-Aldrich), mouse anti-neurofilament (anti-NF) medium chain mAb (NB300-134, 1:500; Novus Biologicals, Littleton, CO, USA), mouse anti-APC (oligodendroglial cells; OP80, 1:100; Calbiochem, San Diego, CA, USA), mouse anti-NeuN (neuronal nuclei) IgG mAb (MAB377, 1:1000; Chemicon) and mouse anti-GM2 ganglioside IgM mAb (mab 10-11, 1:15; cell culture supernatant was produced in-house from the 10-11 hybridoma line by Progenics Pharmaceuticals, Tarrytown, NY, USA). Species-specific secondary antibodies conjugated to Alexa Fluor (AF) 488, 546 and 633 dyes (Invitrogen, Carlsbad, CA, USA) were used for detection of primary antibodies. A minimum of three animals were evaluated for all sex and genotype groups aged 4 and 32-34 weeks. The number of cerebella analyzed in the quantitative studies in 32-week-old animals is listed in the legends to Figs 3,4 and Fig. S1.

### Light microscopy

Overview images (Fig. 1A,B; Fig. 2A,B; Fig. 3A,B,E,F) of the cerebral and cerebellar IF-labeled sections were acquired using an IX70 microscope (Olympus, Tokyo, Japan) equipped with an HQ2 camera (Photometrics, Britannia, AZ, USA) and a Proscan II-encoded *xyz* stage (Prior Scientific, Rockland, MA, USA) equipped with 10 position excitation-emission *Smart*Shutters filter wheels (Sutter, Novato, CA, USA). The exposure time for all antibody combinations was 750 ms (gain 2×) per channel. Excitation/emission conditions for the AF488 and AF546 dyes were excitation (exc.) 490/20/beamsplitter (b.s.) 480-513/emission (em.) 535/40 and exc.572/23/b.s.555-588/em.630/60 nm, respectively. Individual but partly overlapping double-channeled 14-bit images (downsampled to 8-bit) were acquired with a Plan 10× (NA 0.25) objective and digitally stitched in Metamorph/MetaFluor (Molecular Devices, Sunnyvale, CA, USA) and subsequently in Photoshop CS6 (Adobe, San Jose, CA, USA) software. Selected areas of the IF-labeled sections (Figs 1-4; Fig. S1) were imaged by laser scanning confocal microscope (Zeiss Meta Duo V2, Oberkochen, Germany) using a Plan Apochromat 20× (NA 0.8) objective. Conditions of image acquisition (pinhole size, excitation laser intensity, scanning speed, dichroic mirrors, excitation and emission (band)pass filters and photomultiplier gains) were kept constant for individual combinations of primary and secondary antibodies. Excitation and emission filtering was set to minimize crosstalk, and individual dyes were excited and their emission was collected sequentially. The final *z*-stacks (thickness is reported in the legends of the individual figures) were transformed into single-plane maximal intensity projection (MIP) images using the Zeiss LSM Image Browser (Zeiss). Images of amygdala and CA3 brain regions (Fig. 2C-H) represent scans with an open confocal pinhole, allowing collection of the fluorescence information from the entire thickness of the section (35 µm).

### Electron microscopy

For electron microscopy, cerebra of 4- to 5-week-old WT and mutant males and heterozygous females were coronally sectioned (250 µm) using a Vibratome. Sections containing hippocampal CA3 and amygdala regions were selected, and these regions were manually dissected. The tissue blocks were first transferred to 0.1 M cacodylate buffer and post-fixed in 2% glutaraldehyde. Samples were further washed and post-fixed in osmium (1% osmium in 0.1% cacodylate buffer), dehydrated and embedded in Epon-araldite. Ultrathin sections were stained with uranyl acetate and lead citrate and examined with a Philips CM10 electron microscope (Philips Electron Optics, Eindhoven, The Netherlands).

### Purkinje cell density counts

Serial cerebellar sagittal sections (35 µm thick) were visually searched for the first occurrence of the deep cerebellar nuclei (∼0.48 mm lateral; Fig. 105 of Franklin and Paxinos 2008). Such a section was considered as section +1. This and three consecutive sections in lateral sequence (+3, +5 and +7) were stained with a combination of anti-calbindin (PC marker) and anti-β-Gal antibodies and secondary antibodies conjugated to AF488 and AF546, respectively (protocol modified for IF from Praggastis et al., 2015). The total number of Purkinje cells (PC#), the number of PCs with β-Gal-positive nuclei (β-Gal^+^ PCs) and the overall lengths of PC layers (in millimetres) were manually counted/traced in at least three out of the four selected sections in lobules I-II and X. The number of 32-week-old animals analyzed is provided in legends to Figs 3,4 and Fig. S1. Stereo Investigator software (MBF Bioscience, Williston, VT, USA) installed in an Olympus Bx51 microscope was used for outlining the PC layers and counting PCs. PC# and β-Gal^+^ PC# were assessed using a PlanFL 20× (NA 0.5) objective. Although nuclei were co-detected by DAPI in all the double-IF-stained sections, the parallel triple fluorescent quantification proved technically difficult because of unbalanced intensities and fluorescence bleaching of the three dyes. The total number of PCs with structurally identifiable nuclei in lobules I-II and X was first assessed using the fluorescence filter set discerning AF488 (exc.480/40/b.s.510LP/em.505LP). β-Gal^+^ PCs were subsequently quantified by changing the excitation and emission to a triple excitation/emission (DAPI/FITC/TRITC) filter cube (Chroma set #61000v2, Bellows Falls, VT, USA). The overall PC density (PC#/1 mm of the length of the PC layer) and percentage fractions of β-Gal^+^ PCs in individual animals were calculated in lobules I-II and X from values in the 35-µm-thick sections.

### Behavioral studies

The strategy for behavioral testing was designed according to our previous analyses performed in mutant and WT males aged ∼20-24 weeks (Stromme et al., 2011). Expecting a mitigated, delayed and also variable phenotype in heterozygous females compared with the reference abnormality in X-chromosome hemizygous mutant males, we decided to increase substantially the number of tested animals in all compared sex and genotype groups and to perform some of the behavioral tests at an older age (weeks 32-34). Animals were tested for their voluntary locomotor and exploratory activities and anxiety-like (exploration of the center zone) behavior in an open field. Motor coordination was assessed by the balance beam test. Spatial memory of the animals was tested in the object placement test. The timeline of the tests and ages of animals when tested were as follows: balance beam at 20-22 weeks, repeated at 32-34 weeks; object placement at 20-22 weeks; and open field at 32-34 weeks.

Motor coordination was evaluated by counting the number of slips made while crossing a round balance beam (16 mm diameter; 120 cm long; Stanley et al., 2005). Before each testing session, mice were trained to walk over a 6-cm-wide, flat wooden plank to diminish anxiety. Owing to the time scale of the study (8 months) and tissue collection, some animals were tested only once (at either 20-22 or 32-34 weeks old), whereas most were tested longitudinally.

Locomotor activity, exploration and thigmotaxis (anxiety-like behavior) of the animals were assayed in the open-field arena (37 cm×42 cm) for 6 min. The total track length traveled in the entire arena was used as a measure of locomotor activity. Anxiety-like behavior was assessed as the proportion of the center zone (15 cm×15 cm) exploration (center zone track) from the total track traveled. Both measures were assessed automatically using Viewer software (Biobserve, Bonn, Germany). Rears (exploration) were counted manually.

For the object placement test (Ennaceur and Delacour, 1988; Dere et al., 2007), animals were first allowed to explore a pair of identical, non-toxic objects for 5 min (training phase) in an open field with high-contrast visual cues placed on each wall of the arena. Animals were then placed back into their home cages for 16 min (retention interval). Subsequently, one of the objects was moved to a new position, whereas the position of the other object remained unchanged (old position), and the animals were allowed to explore both objects for 3 min (testing phase). Exploration of the objects was defined as any physical contact with an object (whisking, sniffing, rearing on or touching the object) or orienting to the object from within 5 cm. Viewer software (Biobserve) was used to record the sessions. Care was taken to ensure that the intrinsic relationship between the objects and the relative position of the objects to the visual cues was altered. The total times (in seconds) spent exploring the objects in the old and new positions during the testing phase were compared. Animals with cumulative exploration times <2 s in either training or testing phases were excluded from further analyses.

### Statistical analyses and figure preparation

JMP statistical software (v.11; SAS, Cary, NC, USA) was used to analyze the results of the behavioral and PC quantification analyses. The balance beam data were analyzed by a mixed-effects analysis including random and fixed factors, similar to a standard repeated-measures analysis, but with the advantage that missing data can be accommodated in a mixed-effects model that thus analyzed both between (fixed)- and within (random)-subject variability while preserving the correct sample sizes and degrees of freedom (d.f.) when some animals were tested twice (Fig. 5G). The results of the object placement test require analysis of the time exploring the object in both the new and the old position by each mouse (measured during the testing phase). Thus, to preserve the sample size and because the values represent two data points from each individual animal, these data were matched and analyzed by paired *t*-tests in each sex/genotype group. The rest of the statistical analyses (total track lengths, ratios of center and total track lengths, number of rears, total exploration time in the training phase of the object placement test, PC densities and comparisons of fractions of β-Gal^+^ PCs in lobules I-II and X) were evaluated by one-way ANOVA. *P*-values <0.05 were considered statistically significant. Data in Figs 3,4 (shown also as individual values) and 5, in the text and supplementary material, are presented as arithmetic mean values±s.e.m. Figures were prepared in Photoshop CS6 (Adobe, San Jose, CA, USA) and graphs were plotted in GraphPad Prism version 6.05 software (GraphPad Software, La Jolla, CA, USA). For better contrast and visibility without altering the biological information, images were stretched to fill the full dynamic 8-bit ranges.
